# Medicine as a Science or an Art

**Published:** 1896-09-12

**Authors:** 


### Medicine as a Science or an Art.

Medicine has of late held its head so high and posed so self-confidently as a science, that it is well to listen in all due humility to the words of one of its leading professors, who, in speaking of the prognosis of disease—surely that side of medicine which ought to involve the most careful balancing of scientific knowledge—reduces our boasted science to the level of an art, making our decision hinge on the judicious use of well-remembered aphorisms.

The late Sir Russell Reynolds said, "When we can prognosticate with certainty medicine will have become a science," but at present "we deal with doubts and not with certainties," and this may show to us how far medicine is yet from being truly scientific. Professor Allbutt is even harder on medicine as a science, for, speaking of prognosis, he says: "The time when any sufficient rule can be applied to individual cases must long be out of our sight; and the application of any approximate rules must long be subordinate to the instinctive tact of the educated physician himself, who alone can apprehend the sum of the peculiarities which must modify their application to individual instances." This, surely, if expressed in ordinary language, means that medicine is an art rather than a science. What else can be meant by "instinctive tact"?

What, then, had Sir Dyce Duckworth to say on the subject in his address to the British Medical Association at Carlisle? He says that our minds are apt to dwell too much on our detailed notes and our manifold instrumental aids, and too little on the patient; and he draws attention to the study of prognostics, of symptoms which have been found by experience, we know not why, to bear each their own significance.

Let us quote a few of them, culled partly from Hippocrates, as given by Sir Dyce Duckworth. First as regards acute specific diseases we find the following: "Obese and elderly patients are bad subjects for all fevers and acute diseases." "The existence of chronic renal disease is incompatible with recovery from the continued fevers." "Meteorism, delirium, subsultus tendinum, and tremor are always of serious import, indicating failure of nervous power." "The presence of cardiac valvular disease adds greatly to the danger." In typhoid fever dicrotism of the pulse "signifies exhaustion and deep ulceration of the bowel." "Vomiting in the third week may be the precursor of peritonitis." In the case of typhus fever "early epistaxis is an unfavourable symptom." "Coma vigil is always a fatal symptom." "A presentiment of a fatal issue at the outset of a case is commonly a bad sign," &c. All these things are interesting, and if committed to the memory may at times prove to be very useful "tips." But no reasons or explanations are given. True as the aphorisms may be, they are but isolated, unconnected facts, and are as distinct from science as the work of the artist who dabbles in his greens and browns, producing sweet harmonies all the time, although he knows not how, is from that of the scientific chemist who by measured steps alters the formula of his dye stuffs, and so runs through the whole gamut of the colours.

Sir Dyce Duckworth touches on many departments of medicine, and in regard to each he arms his listeners with aphorisms by the aid of which they may be the better able to guess the future and give prognoses which shall not too often fail. While, however, we may admit, readily and freely, that the practitioner armed with these "tips" may often make profound impression on those with whom he may have to deal, and may earn for himself a reputation for prescience which may stand him in good stead, it is not by such means that the science of medicine will be improved nor by such exhibitions that medicine will be able to lay claim even to be a science. When we work with tools the nature of which we know nothing of, we may be artists but certainly we are not scientific.

But the medicine of to-day is something better than this. It cannot be reduced to a mass of aphorisms, and Sir Dyce Duckworth, much stress as he laid on the advantage of always bearing in mind the prognostics which he placed before his hearers, took care to add that "the most accurate prognosis comes from him who has with care, and a large chastened experience, first established a correct diagnosis, and has also learnt to employ remedial measures with judgment and good sense."

